# Stomatoscopes

**Published:** 1884-08

**Authors:** 


### EDITORIAL, ETC.

---

STOMATOSCOPES.—For several years past instruments known as stomatoscopes, intended for illuminating the mouth, especially in cloudy weather and evenings, have been employed and highly recommended by many prominent dentists. The electric light has also been utilized for the same purpose, and also for examinations of the throat, and even of the stomach.

Mr. E. T. Starr, of the S. S. White Dental Manufacturing Company, has quite recently perfected an apparatus concerning which the Philadelphia *Press* speaks as follows:

One of the newest adaptations of electric illumination is in the shape of a very small lantern, which can be introduced into the mouth, throat and (in some cases) the stomach, for the purpose of aiding surgical and dental operations which cannot be carried on without light, and for which it has been extremely difficult heretofore to obtain light by mirrors or other means. Experiments have been making for some years to perfect an apparatus of this kind. Since 1881, E. T. Starr, an expert in electrical science, has been working at intervals upon minute electrical lamps in his rooms, in White's dental establishment, at Twelfth and Chestnut Streets, and at last has succeeded in getting highly satisfactory results. Patents have been obtained. The instrument has been critically examined by dentists and surgeons, and has already been used in practice.

The lamp primarily consists of a delicate glass bulb, from which the air has been withdrawn and as nearly a perfect vacuum created as possible. The bulb varies in shape, being spheroidal, flat and compass-shaped, and also cylindrical, with a conical termination. Through the thin walls of the lantern run the conducting wires, connected by a carbon arc, on which the electricity centers, and which thus becomes the place of light. The glass lantern is very small; the cylindrical-shaped being scarcely half an inch in length and with a diameter not nearly so great as that of an ordinary lead pencil. The compass-shaped lamp is about one quarter of an inch thick and has a diameter of three-quarters of an inch to an inch, while the spheroidal seems scarcely larger than a good-sized pea. The lamp is attached to a handle from seven to nine inches long and about half an inch thick, through which run the wires connecting the battery. The handle on the lamp can be separated, and thus but one handle is necessary for use with the different forms of the lamps. The intensity of the power, and hence the brilliancy of the arc of light, can be regulated by moving along the handle a ring which connects with the wires. The handle has several joints, and its position can be arranged in almost any way so as to adapt it to the shape of the cavity which it is proposed to illuminate. Mirrors can also be fastened to the lamp and light reflected to places where the lamp cannot be introduced. To prevent the too great diffusion of light and the radiation of heat, the lamp may be partially covered with a hard rubber or gutta-percha case.

When the lamp is placed in the mouth of a patient every portion of the throat, even to the lowest parts, and every recess of the upper places can be plainly seen. This will greatly facilitate the work of surgery and dentistry, and enable an operator to conceive a much more thorongh diagnosis of a case than the use of any other means previonsly known. Placed behind the teeth, the intense light renders not only the teeth, but even the gums above, highly transparent. If the teeth are good and undecayed, no lines will be visible; but the presence of a filling or of the mere beginning of decay may at once be seen. When the lamp is placed within the mouth and the lips are closed, the entire front structure of the mouth is brought to view. The bone and tooth formations are easily discoverable, and even the interior of the nasal passages. In the same way the instrument is of great value in the treatment of obstetrical disease, and in studies of the stomach. No unpleasant sensations are experienced by patients, even in cases of protracted use, no other effect being noticeable than that which follows the drinking of a hot cup of coffee.

The difficulties in bringing this application of the electric light to practical use were due principally to the fact that none of the electric manufacturing companies in America could make the right kind of a lamp. Mr. Starr tried them all, and not getting what he wanted in the very best shape, was compelled to go to Europe. More than a score of different lamps, carbons and means of conveying power had to be tried before the best were found.

